# Exploring electronic health record systems implementation in primary health care centres in Saudi Arabia: pre-post implementation

**DOI:** 10.3389/fmed.2025.1502184

**Published:** 2025-06-16

**Authors:** Haitham Alzghaibi, Hayley Hutchings

**Affiliations:** ^1^Department of Health Informatics, College of Applied Medical Sciences, Qassim University, Buraydah, Saudi Arabia; ^2^Swansea University, Medical School, Swansea, United Kingdom

**Keywords:** electronic health records, large-scale project, primary healthcare centers, IT project management, user attitude, Saudi Arabia, Questionnaire

## Abstract

**Background:**

The Saudi government has allocated four billion Saudi Riyals (approximately $1,066 million) to establish the National Electronic Health Record (NEHR) and advance its e-health strategy. Over seventy projects have been identified to achieve this vision. Following the failure of previous initiatives, the Ministry of Health (MoH) in Saudi Arabia is prioritizing the implementation of Electronic Health Record Systems (EHRS) in all Primary Healthcare Centres (PHCs). This study evaluates the implementation of EHRS in PHCs at two phases: pre-implementation and post-implementation.

**Methods:**

A quantitative, self-reported questionnaire was employed at two distinct timescales (pre- and post-implementation) to assess user attitudes and experiences. The study population included all clinical and administrative staff working in Saudi PHCs (*n* = 38,514). A multi-stage cluster sampling technique was used, resulting in data from total 834 participants in both pre and post implementation phases.

**Results:**

Participants demonstrated a high level of awareness regarding the perceived usefulness of EHRS during the pre-implementation phase. In the post-implementation phase, agreement toward EHRS usefulness increased, with 96.6% of participants endorsing the system’s implementation. However, dissatisfaction emerged regarding training and technical support mechanisms. Negative attitudes were also expressed, particularly regarding the time required to assist less experienced users. Variability in user attitudes was observed across scales measuring perceived usefulness, training and support, and negative attitudes. These findings highlight evolving perceptions influenced by direct system use and organizational support.

**Conclusion:**

End-user attitudes toward EHRS implementation vary over time and are influenced by system usability, organizational support, and the scale of the project. Addressing training deficiencies, improving technical support, and involving end-users in the implementation process are critical to fostering positive attitudes and ensuring successful EHRS adoption in PHCs.

## Introduction

The implementation of Electronic Health Record Systems (EHRS) holds the potential to improve care quality and health system efficiency by supporting healthcare professionals in their daily tasks (1–3). Digitising health records through EHRS enhances clinical workflows, minimises administrative burdens, and offers a solid foundation for data-driven decision-making. The effectiveness of these systems is significantly influenced by healthcare professionals’ attitudes and their preparedness to adopt and proficiently utilise the technology. Analysing the factors that affect user acceptance is essential for achieving successful implementation and ensuring long-term sustainability (4, 5).

Research on EHRS adoption has investigated the technical, organisational, and individual factors influencing healthcare professionals’ attitudes (3, 5, 6). Important factors encompass perceived usefulness and ease of use, which notably influence user acceptance (6). Moreover, adequate training, continuous support, and strong leadership during the implementation process are identified as critical elements in promoting favourable user perceptions (5). Organisational factors, including management engagement and support, significantly impact the readiness for EHR adoption (4). Challenges continue to exist, including heightened workloads, time constraints, and restricted user involvement in system design (3, 7). The identified barriers underscore the importance of addressing both technical and organisational aspects to ensure that EHRS are intuitive, efficient, and customised to meet the specific needs of healthcare professionals (1, 3, 8).

The intricate nature of healthcare organisations, characterised by hierarchical structures and varied cultural contexts, highlights the necessity for tailored strategies in the implementation of EHRS (9, 10). The healthcare industry requires solutions that are compatible with its distinct operational and cultural characteristics, unlike other sectors. Findings from different contexts or industries are frequently not directly applicable, especially in culturally specific environments like Saudi Arabia (SA), where organisational, infrastructural, and cultural challenges require careful consideration (11).

The existing literature on EHR adoption predominantly emphasises secondary care organisations or limited projects, resulting in a notable deficiency in insights regarding large-scale implementations within primary healthcare environments (2, 12, 13). Primary Health Centres (PHCs) play a vital role in enhancing the accessibility, accuracy, and security of patient records within community-based care frameworks (1, 14). The incorporation of EHRS in primary healthcare centres enhances care coordination, optimises resource allocation, and improves overall healthcare delivery (9, 15). Nonetheless, there is a paucity of research concerning the organisational factors that affect EHR adoption in PHCs, especially in relation to user readiness, training, and system usability (11).

The implementation of EHRS in SA is a key priority for healthcare policymakers, aligning with the country’s overarching e-health vision and digital transformation objectives (9, 10). The government has designated four billion Saudi Riyals (approximately $1,066 million) for the advancement of the National Electronic Health Record (NEHR) and related e-health initiatives (2, 12, 16). Despite significant investment, challenges remain in the effective implementation of EHRS within the Saudi context, especially in PHCs. Challenges include inadequate user training, insufficient organisational readiness, and resistance to change, all of which impede successful adoption (17).

The Saudi Ministry of Health (MoH) has recognised shortcomings in previous initiatives and has prioritised the implementation of EHRS in PHCs facilities as part of its e-health strategy to rectify past deficiencies and ensure sustainable execution (10). Enhancing the preparedness of PHCs to implement EHRS is essential for meeting national healthcare objectives and contributes to the international dialogue on the application of EHRS in culturally unique and resource-constrained environments. This study aims to evaluate EHRS implementation in PHCs, concentrating on user readiness, training, and system usability, to offer actionable insights for policymakers and healthcare administrators in SA and beyond.

This study seeks to address the knowledge gap by assessing the implementation of EHRS in PHCs in SA from an organisational viewpoint. This study examines user readiness, training and support mechanisms, as well as the perceived usability and effectiveness of EHRS. The findings will enhance the existing evidence on EHRS implementation and offer practical insights for policymakers and healthcare administrators in SA and comparable healthcare systems worldwide.

### Aim

This study aims to evaluate the implementation of the EHRS in the PHCs in two different phases (pre and post implementation).

## Methods

### Study design

This study employed a cross-sectional design to evaluate end-user perspectives on EHRS implementation at two distinct phases: pre-implementation and post-implementation. Data were collected at each phase using a structured, self-administered questionnaire to assess user attitudes, readiness, and experiences. The cross-sectional approach facilitated the comparison of user perceptions at two specific time points, providing insights into the immediate impacts of EHR implementation. This design enables the identification of factors influencing the adoption process and highlights areas requiring improvement to optimize EHR integration in PHC service delivery.

### Population and sampling technique

The study population comprised clinical staff including physicians, nurses, pharmacists, laboratory technicians, and dentists as well as administrative personnel, such as managers, secretaries, and receptionists, all of whom were employed at PHCs (PHCs) across SA (*N* = 38,514) (18). The practitioners came from diverse backgrounds, including different professional roles, age groups, and genders. A multi-stage cluster sampling technique was employed to ensure broad geographic representation in both the pre- and post-implementation phases of the study.

**Figure 1:**
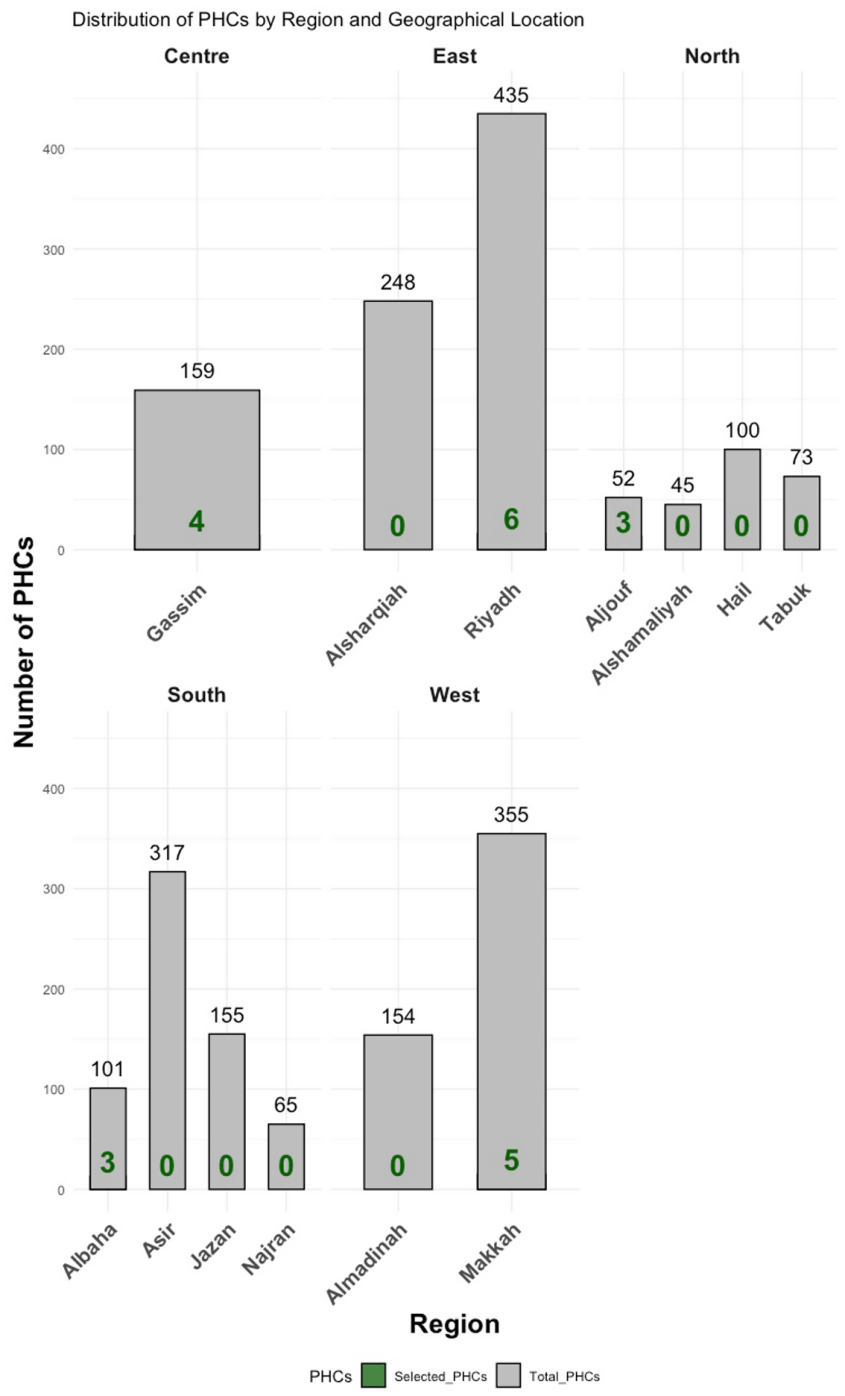
Main regions in SA and the number of selected PHCs in each region.

- In Stage One, the researcher adopted the Ministry of Health’s administrative division of SA into thirteen regions, treating each as a cluster (see Figure 1).
- In Stage Two, five representative regions were selected using simple random sampling, with consideration given to geographic diversity for example, Makkah was chosen to represent the western region, and Albaha the southern region.
- In Stage Three, 21 PHCs were randomly selected from these five regions. From these centres, a random sample of 2,259 eligible staff was drawn for participation.

Of those invited, 351 individuals completed the questionnaire during the pre-implementation phase, and 483 different individuals responded during the post-implementation phase, resulting in a combined total of 834 respondents across both phases. Due to logistical and practical constraints such as staff turnover, availability, and anonymised data collection it was not possible to survey the same individuals in both phases. Therefore, the study employed a repeated cross-sectional design rather than a cohort design, allowing for comparison of group-level changes in attitudes and readiness between the two time points.

### Inclusion and exclusion criteria

The study included clinical and administrative personnel from 21 PHCs (PHCs) selected from five out of SA’s 13 administrative regions, based on a multi-stage cluster sampling approach. The reference to 21 PHCs pertains to the total number of individual healthcare facilities selected for data collection not to administrative clusters. All selected PHCs were governmental facilities operating under the Ministry of Health. Eligible participants included physicians, nurses, pharmacists, laboratory technicians, and administrative staff such as receptionists and managers. To be included in the study, participants were required to be actively employed at one of the selected PHCs during the data collection period and to hold roles either directly or indirectly related to the implementation or use of the Electronic Health Record System (EHRS). Exclusion criteria included practitioners working in non-governmental facilities (e.g., private PHCs), secondary or tertiary care institutions, dental clinics, and independent laboratories. Additionally, temporary or outsourced staff not engaged in EHRS-related tasks, and individuals unwilling or unable to provide informed consent, were excluded from the study.

The sampling approach was designed to capture a broad range of perspectives from all staff categories involved in EHRS use, whether directly or indirectly. Administrative staff such as receptionists and managers although less involved in clinical documentation, play essential roles in patient registration, appointment scheduling, and information handling, all of which interface with the EHRS. Therefore, their inclusion was intentional to reflect the comprehensive nature of system use within PHCs.

### Questionnaire development

The methods employed in this investigation was the use of a structured, self-administered questionnaire consisting of predetermined items and response choices. After careful examination, it was determined that the literature questionnaires pertaining to the topic were prevalent and more efficient compared to other approaches in evaluating the preparedness and knowledge of healthcare personnel in implementing an EHRS (13, 19–23). Prior research has indicated that individual readiness evaluations can be undertaken by considering seven criteria: computer skills, gender, attitudes toward the implementation of the EHRS, knowledge about EHRS implementation and experience at work, age (23–26), hence, the purpose of the second portion was to gather the participants’ demographic information. The second set of questions (*n* = 13) in the third segment assessed the preparedness of the PHC facilities for implementation, as perceived by the PHC personnel. The questions utilized in this section were derived from a pre-existing survey known as the “Organisational Information Technology/Systems Innovation Readiness Scale (OITSIRS).” (27). Both scales are intended to collect responses using a 5-point Likert scale that spans from Strongly disagree to Strongly agree. The scale is as follows: Strongly disagree (1); Disagree (2); Agree (3); Strongly agree (4); and No opinion (5). In addition, a total of 37 items were extracted from a pre-existing questionnaire known as “The Clinical Information System Implementation Evaluation Scale (CISIES)” (28), to assess user attitudes toward the implementation of EHRS (before and after). Additionally, the questionnaire included a fourth segment with open-ended questions designed to gather feedback and insights from participants, enabling a richer understanding of their perspectives and recommendations regarding EHRS implementation.

The validity of the questionnaire has been assessed over two rounds. During phase one, the initial version of the questionnaire was distributed to a group of experts for their review. The purpose of this review was to ensure that all questions could be answered by the intended audience and to prevent any irrelevant responses that could potentially impact the reliability of the questionnaire. Phase Two: After receiving input and suggestions from expert panels, the questionnaire was distributed to a limited number (*n* = 13) of end-users of the deployed EHRS in Saudi PHCs as pilot study. The purpose of this pilot study was to verify that the questionnaire was easily comprehensible, unambiguous, and dependable.

### Data collection procedures

This study’s data collection involved distributing an online, self-administered questionnaire through surveymonkey.com to participants in 21 selected PHCs in SA. This method was selected based on various practical considerations. The extensive geographical area of SA rendered the physical distribution of paper-based questionnaires impractical, particularly due to the limited postal services available. Furthermore, the substantial sample size would have made paper-based distribution expensive and logistically complex. The remote nature of the study facilitated efficient and accessible data collection through an online format.

The distribution of the questionnaire occurred over ten weeks in pre-implementation and eight weeks in post-implementation phase. In both phases participants received notifications via representatives from the selected PHCs, who were supplied with ethical approval documents and invited to join a designated WhatsApp group. Upon joining the group, representatives obtained a distinct survey link created through surveymonkey.com, which they disseminated to their colleagues through their emails and various communication channels, including internal WhatsApp groups. Two reminder messages were sent in each phase of data collection to improve the response rate. This method promoted extensive distribution and encouraged involvement throughout the chosen PHCs.

### Data analysis

The data were analysed utilizing SPSS V.22. Initially, the study’s scales underwent a reliability test using Cronbach’s alpha to verify the consistency of the data gathering tool. Subsequently, a descriptive analysis was conducted to present the data collected from the participants. This analysis involved employing measures such as median, percentages, complete agreement, and rank. The provided replies were utilized to compute a cumulative agreement score for each individual question. The questions were ranked according to the degree of consensus, ranging from the highest to the lowest level of agreement. The total agreement was consolidated by combining the responses of “agree” and “strongly agree.”

Following the presentation of descriptive statistics, inferential analyses were conducted to explore group-level differences and associations within the dataset. Given the ordinal and nominal nature of the variables, non-parametric tests were employed. Specifically, the Mann–Whitney U test was used to compare differences between two independent groups, while the Kruskal–Wallis test was applied for comparisons involving three or more groups. These tests were used to examine the influence of demographic factors such as age, gender, professional experience, and occupation on participants’ readiness for EHRS implementation. This approach enabled a rigorous assessment of the relationships between participant characteristics and readiness levels, aligned with the distributional properties of the data.

### Responses to open-ended questions

Responses to the open-ended questions were presented and organised using Microsoft Excel. All responses were exported as text from surveymonkey.com to a Microsoft Excel sheet. Arabic responses were translated to English by the author and then checked by a professional translator. Thematic analysis approach was utilised to analyse responses of open-ended questions which were then grouped into themes by the where similar responses were gathered under one theme. All themes were then coded to label each response with the appropriate code to allow Excel to calculate the number of responses in each theme.

## Results

The study assessed the reliability of the scales using Cronbach’s Alpha, which indicated high internal consistency across all constructs (Table 1). Pre-implementation scales exhibited high reliability, indicated by a Cronbach’s Alpha of 0.94 for PHC staff awareness regarding the perceived usefulness of the EHRS and 0.89 for PHC readiness. The post-implementation scales demonstrated high reliability, indicated by Cronbach’s Alpha values of 0.92 for perceived usefulness of the EHRS, 0.89 for positive attitude, 0.90 for training and support, and 0.85 for negative attitude. The results indicate that the scales demonstrate high reliability for assessing the intended constructs during both pre- and post-implementation phases.

**Table 1:** Scale reliability using Cronbach’s alpha.

Scale	Number of Items	Cronbach’s Alpha
Pre-implementation		
PHCs staff awareness about perceived usefulness of EHRS	13	0.94
PHCs readiness	13	0.89
Post-implementation		
Perceived usefulness of the EHRS	13	0.92
Positive attitude	15	0.89
Training and support	7	0.90
Negative attitude	7	0.85

### Pre-implementation

**Figure 2:**
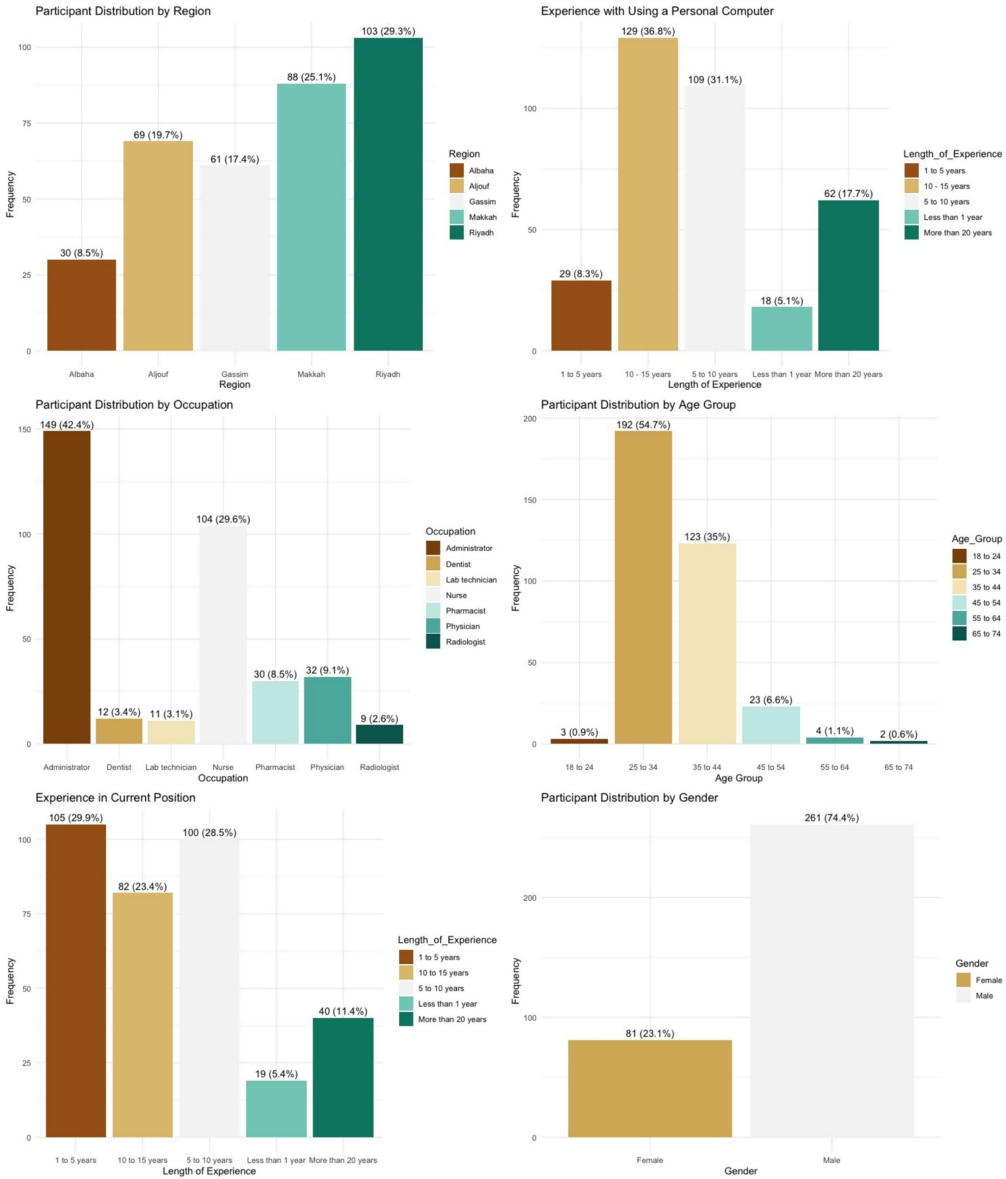
Participant demographic distribution: pre-implementation.

The questionnaire data were collected from 351 participants across five different regions of SA. The largest number of the respondents, 103 (29.3%), were residents of the capital city, Riyadh (see Figure 2). All participants worked in healthcare and administrative roles. As can be seen in Figure 2, 149 (42.4%) were in an administrative role such as managers, secretaries and receptionists; 104 (29.6%) worked in a nursing role; thirty-two (9.1%) were physicians; and thirty (8.5%) were pharmacists. Four (1.1%) participants did not declare their occupation.

Age was measured via six categories, as illustrated in 8 below. The majority of participants, 192 (54.7%), were between twenty-five and thirty-four years of age. A detailed breakdown of the age categories is provided in 8. Four (1.1%) participants did not declare their age. Participants were also asked to specify their gender. Participants were mostly male (*n* = 261; 74.4%). Out of 351 participants, only eighty-one (23.1%) were female. Nine (2.6%) participants did not declare their gender.

Participants were asked to report their personal experience using a computer at home. The majority (*n* = 129; 36.8%) indicated between ten and fifteen years of experience, while a small group (*n* = 18; 5.1%) reported less than one year. Four participants (1.1%) did not respond to this question. A detailed breakdown is presented in Figure 2.

Participants were also asked how long they had been employed in their current role. The largest group (*n* = 105; 29.9%) reported one to five years of experience. Five participants (1.4%) did not provide a response to this item. A full distribution is illustrated in Figure 2.

### Participant perceptions of the perceived usefulness of an EHRS

In order to determine the participant awareness and perceptions of the usefulness of an EHRS, they were asked to answer thirteen items reflecting the possible benefits of an EHRS. All the items generated high agreement scores, ranging from 90.5 to 84.2%. This clearly indicates that most of the participants agree with all the statements on the questionnaire. It was observed that the highest ranked benefit was “*Information from the EHRS enables me to make better decisions about patient care”* (90.5%), followed by “*EHRS provides accurate, up-to-date and complete information about patients at the point of care*” (89.9%), “*EHRS enable quick access to patient records for more coordinated, efficient care*” (89.5%), and “*EHRS improve patient and healthcare professionals interaction and communication as well as healthcare convenience*” (89.0%). At the bottom of the scale, though still with high agreement, the least ranked items were “*EHRS improve end-users’ productivity and efficiency*” (87.3%), *“EHRS enable safer, more reliable prescribing”* (87.0%), “*EHRS improve the privacy and security of patient data*” (85.8%) and, finally, “*Using the EHRS helps to reduce medical errors*” (84.2%). Overall, participants express high level of awareness about the perceived usefulness of the EHRS (see Table 2).

**Table 2:** Participant responses to statements regarding PHC staff awareness of the perceived usefulness of an EHRS (*N* = 13).

Items		Strongly Disagree (1)	Disagree (2)	Neutral (3)	Agree (4)	Strongly Agree (5)	Median	Total agreement	Rank
Information from EHRS enables better decisions about patient care	N	22	8	3	154	160	4	314	1
	%	6.3	2.3	0.9	44.4	46.1		90.5	
EHRS provide accurate, up-to-date and complete information about patients at the point of care	N	19	13	3	160	150	4	310	2
	%	5.5	3.8	0.9	46.4	43.5		89.9	
EHRS enables quick access to patient records for more co-ordinated, efficient care	N	23	9	4	123	184	5	307	3
	%	6.7	2.6	1.2	35.9	53.6		89.5	
EHRS improves patient and healthcare professionals’ interaction and communication as well as healthcare convenience	N	24	8	6	122	185	5	307	4
	%	7.0	2.3	1.7	35.4	53.6		89.0	
Using EHRS helps to effectively diagnose patients	N	21	15	4	137	169	4	306	5
	%	6.1	4.3	1.2	39.6	48.8		88.4	
EHRS reduces costs through decreased paperwork, improved safety, reduced duplication of testing and improved health	N	24	12	4	108	195	5	303	6
	%	7.0	3.5	1.2	31.5	56.9		88.4	
EHRS helps to promote legible, complete documentation and accurate, streamlined coding and billing.	N	24	12	5	113	191	5	304	7
	%	7.0	3.5	1.4	32.8	55.4		88.2	
Sharing electronic information with patients and other clinicians is more secure when using an EHRS	N	24	15	4	134	167	4	301	8
	%	7.0	4.4	1.2	39.0	48.5		87.5	
An EHRS helps to provide safer care	N	23	16	5	128	174	5	302	9
	%	6.6	4.6	1.4	37.0	50.3		87.3	
EHRS improves end-user productivity and efficiency	N	24	15	5	121	180	5	301	10
	%	7.0	4.3	1.4	35.1	52.2		87.3	
EHRS enables safer, more reliable prescribing.	N	22	15	8	109	191	5	300	11
	%	6.4	4.3	2.3	31.6	55.4		87.0	
EHRS improves the privacy and security of patient data	N	22	22	5	110	185	5	295	12
	%	6.4	6.4	1.5	32.0	53.8		85.8	
EHRS helps to reduce medical errors	N	25	22	7	127	161	4	288	13
	%	7.3	6.4	2.0	37.1	47.1		84.2	

### Participant responses regarding perceived readiness for EHRS implementation

This section assesses the perceived preparedness of PHCs for the implementation of EHRS, drawing on participant responses to 13 critical items. The findings underscore strengths and challenges in readiness.

Items associated with positive attitudes toward EHRS adoption exhibited the highest level of agreement. Among participants, 84.1% expressed agreement or strong agreement regarding the positive attitude toward EHRS implementation, identifying it as the most supported readiness factor. Subsequently, there was an 82.5% agreement regarding users’ general support for EHRS, an 82.2% agreement on their willingness to participate in the implementation process, and a 74.1% agreement on users possessing an adequate level of computer literacy. The results indicate a positive perception of user readiness for the adoption of EHRS in PHCs.

In contrast, aspects concerning communication and user involvement exhibited notable disagreement. Only 22.2% of participants indicated agreement or strong agreement regarding the existence of formal communication mechanisms to facilitate interactions between users and IT support staff. In a similar vein, only 19.8% concurred that sufficient communication mechanisms exist to facilitate shared communication across all organisational levels. Additionally, merely 18% of participants reported that staff were involved in decision-making processes. The findings indicate deficiencies in communication infrastructure and staff engagement during the implementation process.

The item with the least endorsement pertained to the adequacy of current work practices, with merely 15.4% of respondents agreeing that existing information systems sufficiently support current workflows. Additional low-ranking factors comprised the presence of a core user group (23.2%), awareness of EHRS utilisation by other organisations (23.0%), and the focus on interdisciplinary collaboration (24.5%) (see Table 3).

**Table 3:** Participant responses regarding the perceived readiness of PHCs to implement an EHRS (*N* = 13).

Items		Strongly Disagree (1)	Disagree (2)	Neutral (3)	Agree (4)	Strongly Agree (5)	Median	Total agreement	Rank
Individuals have a positive attitude toward EHRS implementation	N	19	30	5	116	171	5	287	1
	%	5.6	8.8	1.5	34.0	50.1		84.1	
Users are typically supportive of an EHRS	N	18	39	4	164	122	4	286	2
	%	5.2	11.2	1.2	47.3	35.2		82.5	
There is a willingness to engage in the EHRS implementation process	N	23	32	6	114	168	4	282	3
	%	6.7	9.3	1.7	33.2	49.0		82.2	
Most users have an adequate level of computer literacy	N	27	59	4	160	97	4	257	4
	%	7.8	17.0	1.2	46.1	28.0		74.1	
Staff are typically involved in EHRS implementation.	N	72	140	19	77	36	2	113	5
	%	20.9	40.7	5.5	22.4	10.5		32.9	
Adequate training is available to support users	N	75	145	16	60	49	2	109	6
	%	21.7	42.0	4.6	17.4	14.2		31.6	
There is an emphasis on the importance of collaborative interdisciplinary teams to support EHRS implementation	N	89	149	21	55	29	2	84	7
	%	25.9	43.4	6.1	16.0	8.5		24.5	
A core group of users (champions) is available to support implementation	N	85	154	26	52	28	2	80	8
	%	24.6	44.6	7.5	15.1	8.1		23.2	
Knowledge about how EHRS is being used by other organisations is available	N	92	158	15	55	24	2	79	9
	%	26.7	45.9	4.4	16.0	7.0		23.0	
Formal communication mechanisms exist to support user and IT support staff communication	N	97	150	19	49	27	2	76	10
	%	28.4	43.9	5.6	14.3	7.9		22.2	
Adequate communication mechanisms exist to support shared communication across all organisational levels	N	106	148	22	48	20	2	68	11
	%	30.8	43.0	6.4	14.0	5.8		19.8	
Staff have been included in decision-making processes	N	111	153	17	43	19	2	62	12
	%	32.4	44.6	5.0	12.5	5.5		18	
Current work practices are adequately supported by existing information systems	N	115	154	23	32	21	2	53	13
	%	33.3	44.6	6.7	9.3	6.1		15.4	

### Participant responses regarding endorsement of EHRS implementation

**Figure 3:**
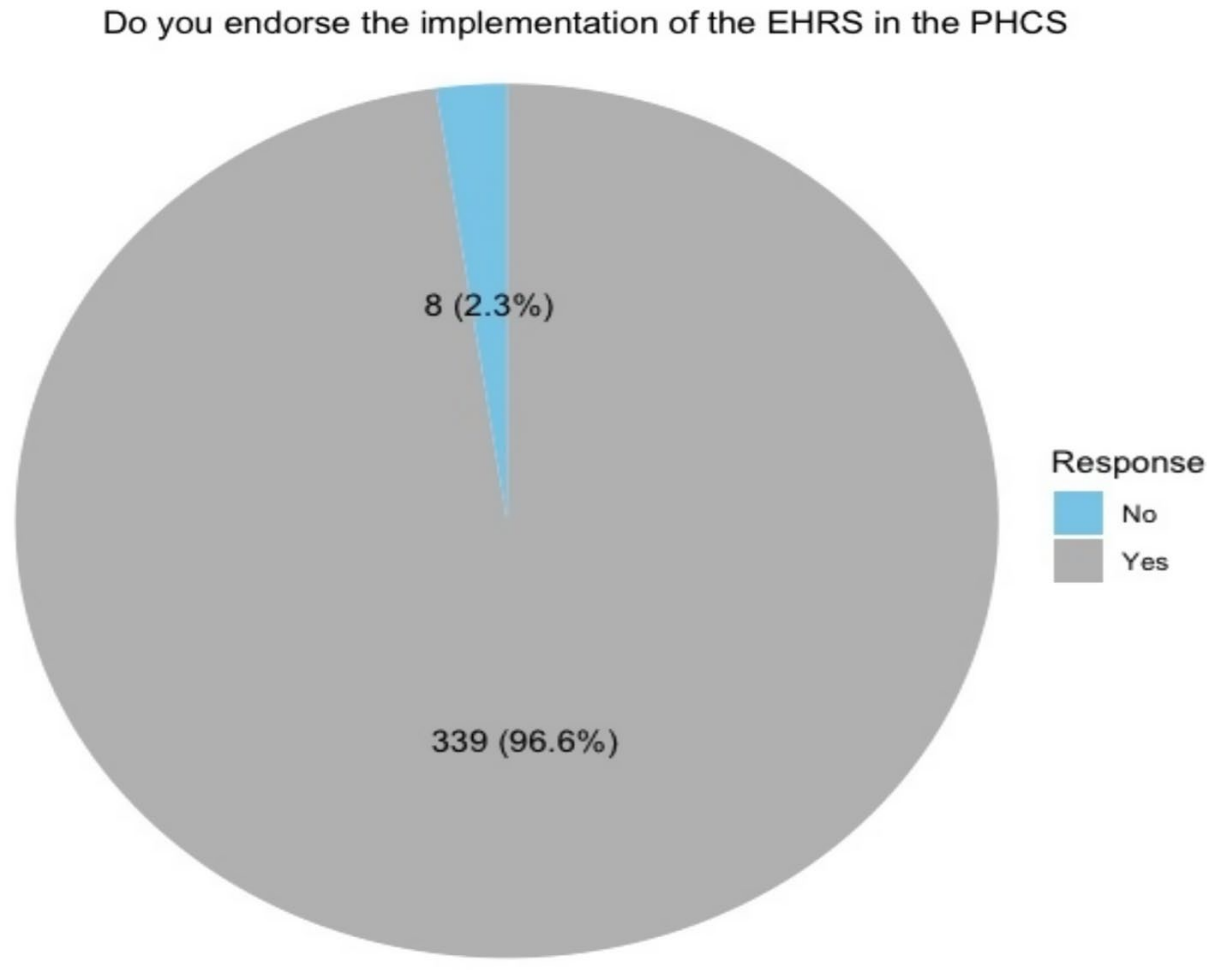
Do you endorse the implementation of the EHRS in the PHCS.

The majority of participants (96.6%, *n* = 339) expressed support for the endorsement of EHRS implementation in PHCs, whereas a minority (2.3%, *n* = 8) indicated opposition. Furthermore, 1.1% (*n* = 4) of participants did not indicate their position on the implementation of EHRS (see Figure 3).

### Inferential statistics

This section presents the results of statistical analyses assessing the impact of demographic and experiential factors on PHC staff perceptions regarding EHRS implementation. Given the ordinal nature of the data, Mann–Whitney U and Kruskal-Wallis tests were conducted to identify any significant differences across participant groups.

A Mann–Whitney U test was performed to examine gender differences in perceptions of EHRS. The results indicated no significant differences between male and female participants regarding PHC staff awareness of EHRS usefulness (*p* = 0.506), readiness for EHRS adoption (*p* = 0.344), staff resistance (*p* = 0.079), or willingness to use EHRS (*p* = 0.925) (Table 4).

**Table 4:** Mann–Whitney U test for gender differences.

Variable	PHC staff awareness of EHRS usefulness	Readiness for EHRS	Staff willingness
Gender	0.506	0.344	0.925

A Kruskal-Wallis test was conducted to examine whether demographic and experiential factors influenced participants’ perceptions of EHRS across multiple scales, including perceived usefulness, readiness, resistance, and willingness to adopt EHRS. The results indicated that occupation had no significant impact on perceptions, with *p*-values exceeding 0.05 across all scales (usefulness: *p* = 0.450, readiness: *p* = 0.475, resistance: *p* = 0.441, willingness: *p* = 0.467). Similarly, age did not significantly influence responses, with no meaningful differences detected for usefulness (*p* = 0.074), readiness (*p* = 0.616), or willingness (*p* = 0.055). Given the small number of participants in some age categories, a Mann–Whitney U test was performed for the two most populated groups (25–34 and 35–44), but no significant differences were found.

Further analysis assessed the impact of experience using personal computers, but no significant variations were observed across the three levels of computer experience, with *p*-values of 0.757 for usefulness, 0.925 for readiness, and 0.146 for willingness. Likewise, years of job experience did not significantly affect EHRS perceptions, with all scales showing non-significant results (usefulness: *p* = 0.771, readiness: *p* = 0.984, willingness: *p* = 0.529). Lastly, the effect of province of residence was examined to determine whether regional differences influenced perceptions. However, the results revealed no significant impact on any of the measured variables (usefulness: *p* = 0.283, readiness: *p* = 0.853, willingness: *p* = 0.145), suggesting that location did not play a determining role in participants’ views on EHRS adoption.

These findings indicate that none of the examined demographic or experiential factors significantly influenced PHC staff perceptions of EHRS adoption, reinforcing the notion that attitudes toward EHRS may be shaped by other, more nuanced factors beyond basic demographic attributes. Future research may explore organizational culture, training programs, and system usability to gain deeper insights into EHRS adoption drivers (see Table 5).

**Table 5:** Kruskal-Wallis test for occupation, age, computer experience, job experience, and province differences.

Variable	PHC staff awareness of EHRS usefulness	Readiness for EHRS	Staff willingness
Occupation	0.450	0.475	0.467
Age	0.074	0.616	0.055
Experience with PCs	0.757	0.925	0.146
Job experience	0.771	0.984	0.529
Province	0.283	0.853	0.145

### Post-implementation

**Figure 4:**
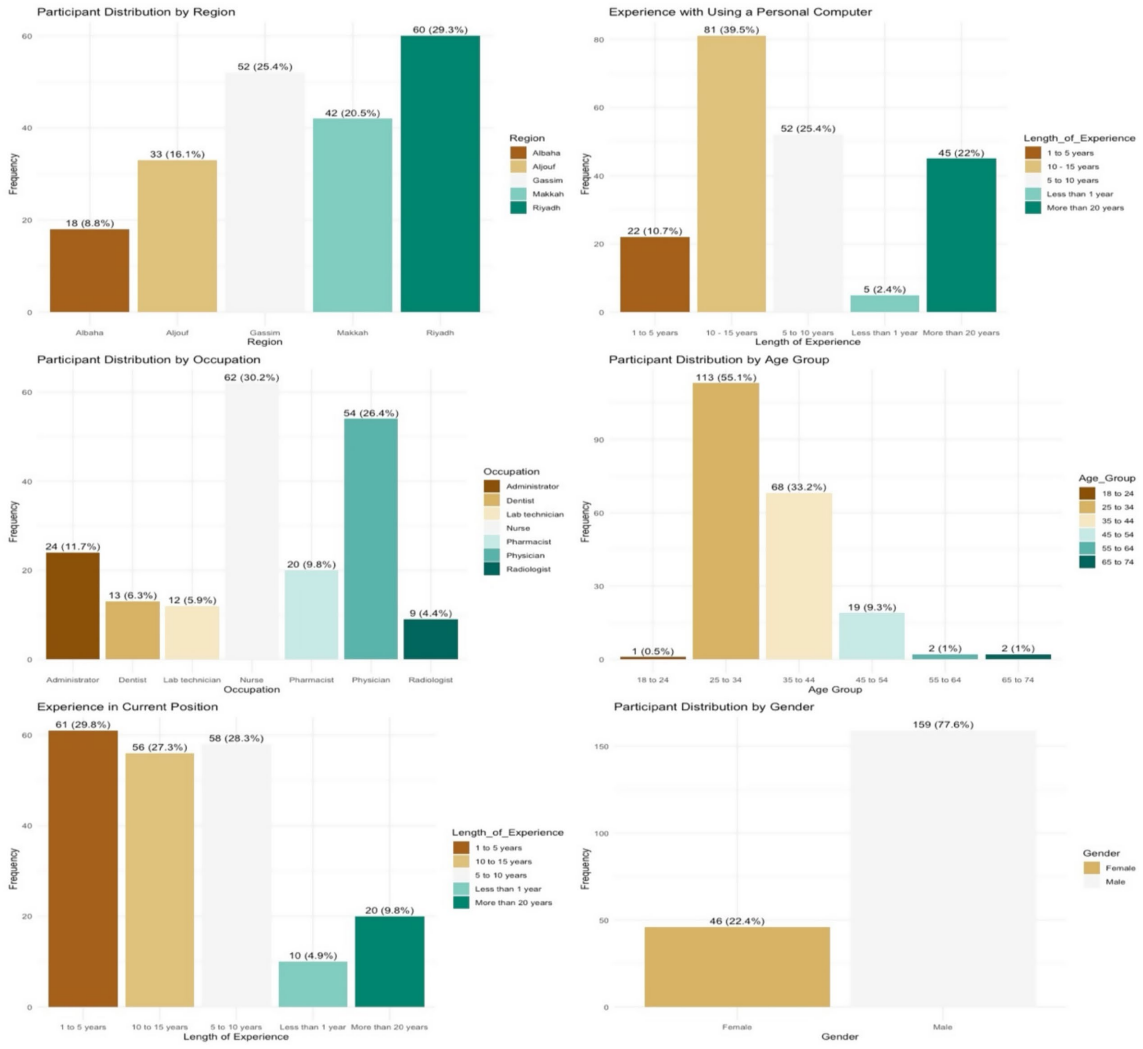
Participant demographic distribution: post-implementation.

A total of 205 healthcare practitioners completed the questionnaire during the post-implementation phase, yielding a response rate of 42.4% from the 483 practitioners invited. The highest proportion of responses originated from Riyadh (*n* = 60; 29.3%), followed by Qassim (*n* = 52; 25.4%), Makkah (*n* = 42; 20.5%), Aljouf (*n* = 33; 16.1%), and Albaha (*n* = 18; 8.8%) (see Figure 4).

The participant group was predominantly male (*n* = 159; 77.6%), while female respondents accounted for 22.4% (*n* = 46). This gender distribution reflects the broader national workforce structure in SA, where clinical roles are predominantly occupied by male professionals, and female representation is concentrated in nursing roles, many of which are filled by expatriate staff from Southeast Asia (see Figure 4).

Respondents represented a variety of healthcare occupations. The largest group were nurses (*n* = 62; 32%), followed by administrative staff (*n* = 54; 26.4%), physicians (*n* = 24; 12.4%), and pharmacists (*n* = 20; 10.3%). Eleven participants (5.4%) did not disclose their occupational role. This occupational breakdown differs from the pre-implementation phase, where a greater proportion of respondents were administrative staff (42.4%), likely reflecting differences in availability or response dynamics between phases.

In terms of age, the majority of participants (*n* = 113; 55.1%) were between 25 and 34 years old. Regarding professional tenure, 61 participants (29.8%) had worked in their current role for between one and five years, 58 (28.3%) for six to ten years, and 56 (27.3%) for eleven to fifteen years. Only 20 participants (9.8%) reported more than fifteen years of experience, and 10 (4.9%) had less than one year of experience. Participants were also asked about their experience using a personal computer at home. The findings indicated that the majority had more than five years of computer use experience, suggesting a relatively high level of digital literacy among the study population (see Figure 4).

### Perceived usefulness of the EHRS

This section assesses participants’ perceptions regarding the utility of an EHRS, derived from responses to 17 items in the questionnaire. Participants demonstrated a high degree of satisfaction regarding the utility of the implemented EHRS. Agreement levels for all items varied between 93.7 and 87.3%, indicating robust positive perceptions regarding the system’s advantages.

The statement indicating that the EHRS reduces costs through decreased paperwork, enhanced safety, reduced duplication of testing, and improved healthcare received the highest level of agreement at 93.7%. The EHRS demonstrated significant utility in enhancing document legibility (93.1%), securely sharing electronic information with patients and clinicians (92.7%), and facilitating streamlined coding processes (92.7%).

Additional areas of significant consensus encompassed the EHRS facilitating rapid access to patient records for enhanced coordination and efficiency in care (92.2%), enhancing interaction and communication between patients and healthcare professionals (92.2%), and permitting increased focus on other dimensions of patient care (91.8%). Participants acknowledged the contribution of EHRS in delivering accurate information (91.7%), facilitating safer and more reliable prescribing (91.2%), and aiding comprehensive documentation (90.3%).

Nonetheless, certain items exhibited comparatively lower levels of agreement, while still indicating positive perceptions. The EHRS demonstrated capabilities in enhancing patient data privacy and security (88.7%), delivering safer care (88.7%), facilitating improved decision-making in patient care (88.3%), and aiding effective diagnosis (88.2%). The statement that the EHRS contributes to a reduction in medical errors received the lowest endorsement at 87.3% (see Table 6).

**Table 6:** Degree of endorsement for each of the seventeen questions relating to perceived usefulness of EHRS.

Items		Strongly Disagree (1)	Disagree (2)	Neutral (3)	Agree (4)	Strongly Agree (5)	Median	Total agreement	Rank
EHRS reduces costs through decreased paperwork, improved safety, reduced duplication of testing and improved healthcare	N	5	4	4	87	105	5.00	192	1
	%	2.4	2.0	2.0	42.4	51.2		93.6	
EHRS help to promote legible documents	N	4	6	4	94	95	4.00	189	2
	%	2.0	3.0	2.0	46.3	46.8		93.1	
Sharing electronic information with patients and other clinicians is more secure when using the EHR system	N	6	4	5	91	99	4.00	190	3
	%	2.9	2.0	2.4	44.4	48.3		92.7	
The EHR system helps with streamlined coding	N	4	6	5	97	93	4.00	190	4
	%	2.0	2.9	2.4	47.3	45.4		92.7	
EHRS enable quick access to patient records for more coordinated and efficient care.	N	8	5	3	95	94	4.00	189	5
	%	3.9	2.4	1.5	46.3	45.9		92.2	
Using the EHR system improves patient and healthcare professionals’ interaction and communication as well as healthcare convenience	N	5	5	6	101	88	4.00	189	6
	%	2.4	2.4	2.9	49.3	42.9		92.2	
The EHR system allows me to spend more time on other aspects of patient care	N	6	6	5	94	94	4.00	188	7
	%	2.9	2.9	2.4	45.9	45.9		91.8	
EHRS help to provide accurate information	N	5	7	5	100	88	4.00	188	8
	%	2.4	3.4	2.4	48.8	42.9		91.7	
EHRS enable safer and more reliable prescribing	N	4	6	8	88	99	4.00	187	9
	%	2.0	2.9	3.9	42.9	48.3		91.2	
EHRS help to have complete documentation	N	6	8	6	92	93	4.00	185	10
	%	2.9	3.9	2.9	44.9	45.4		90.3	
EHRS provide accurate, up-to-date and complete information about patients at the point of care	N	5	8	7	106	79	4.00	185	11
	%	2.4	3.9	3.4	51.7	38.5		90.2	
EHRS improve end-user productivity and efficiency	N	6	9	7	90	92	4.00	182	12
	%	2.9	4.4	3.4	44.1	45.1		89.2	
EHRS improve the privacy and security of patient data	N	6	6	11	88	93	4.00	181	13
	%	2.9	2.9	5.4	43.1	45.6		88.7	
Using the EHR system helps to provide safer care	N	4	8	11	93	88	4.00	181	14
	%	2.0	3.9	5.4	45.6	43.1		88.7	
Information from the EHR system enables me to make better decisions about patient care	N	5	8	11	99	82	4.00	181	15
	%	2.4	3.9	5.4	48.3	40.0		88.3	
Using the EHR system helps to effectively diagnose patients	N	5	9	10	96	83	4.00	179	16
	%	2.5	4.4	4.9	47.3	40.9		88.2	
Using the EHR system helps to reduce medical errors	N	6	7	13	96	83	4.00	179	17
	%	2.9	3.4	6.3	46.8	40.5		87.3	

### PHCs staff attitudes toward use of the EHRS

As seen in Table 7 positive attitudes toward EHR system (EHRS) implementation and use indicated a significant level of overall endorsement, with agreement rates between 79.6 and 97.5%. The highest levels of agreement were noted for: (1) a preference for EHRS over paper-based systems (97.5%), (2) the perceived efficiency of EHRS relative to paper-based systems (95.1%), and (3) its role in enhancing adherence to policies and procedures (92.6%). In contrast, the lowest levels of agreement, though still reflecting general positivity, were noted for: (13) addressing the specific needs of care areas (81.9%), (14) the overall effectiveness of EHRS introduction (80%), and (15) commitment to the system’s successful use (79.6%).

**Table 7:** PHCs staff attitudes towards use of the EHRS.

Items		Strongly Disagree (1)	Disagree (2)	Neutral (3)	Agree (4)	Strongly Agree (5)	Median	Total agreement	Rank
Positive attitude									
Overall, I prefer using the EHR system to the paper-based system	N	1	2	2	91	107	4	198	1
	%	0.5	1.0	1.0	44.8	52.7		97.5	
The EHR system is more efficient than a paper-based system	N		4	6	80	113	4	193	2
	%		2.0	3.0	39.4	55.7		95.1	
I can depend on the accuracy of the EHR system	N	1	6	6	104	85	4	189	3
	%	0.5	3.0	3.0	51.5	42.1		93.6	
Using EHRS leads to better adherence to policies and procedures	N	3	5	7	108	81	4	189	4
	%	1.5	2.5	3.4	52.9	39.7		92.6	
The EHR system facilitates the communication of patient information among members of our healthcare team.	N	2	4	13	87	99	4	186	5
	%	1.0	2.0	6.3	42.4	48.3		90.7	
I am physically comfortable while using the EHR system equipment and hardware	N	4	8	14	90	88	4	178	6
	%	2.0	3.9	6.9	44.1	43.1		87.2	
The EHR system has improved my practice	N	4	6	21	78	96	4	174	7
	%	2.0	2.9	10.2	38.0	46.8		84.8	
I feel the use of the EHR system has improved the quality of patient care	N	2	7	21	99	75	4	174	8
	%	1.0	3.4	10.3	48.5	36.8		85.3	
The EHR system is easy to use	N	3	7	18	104	70	4	174	9
	%	1.5	3.5	8.9	51.5	34.7		86.2	
I feel the use of the system has improved patient care outcomes	N	5	5	25	84	86	4	170	10
	%	2.4	2.4	12.2	41.0	42.0		83.0	
Information almost never gets lost in the EHR system	N	5	11	18	84	85	4	169	11
	%	2.5	5.4	8.9	41.4	41.9		83.3	
The EHR system takes into account the specific needs of my care area(s)	N	6	10	21	90	78	4	168	12
	%	2.9	4.9	10.2	43.9	38.0		81.9	
Overall, the introduction of the EHR system has been effective	N	8	9	24	79	85	4	164	13
	%	3.9	4.4	11.7	38.5	41.5		80.0	
I am committed to the successful use of the EHR system	N	12	7	23	86	77	4	163	14
	%	5.9	3.4	11.2	42.0	37.6		79.6	
Attitudes toward training and support									
I do not get as much help as I need to fix problems with EHRS	N	14	19	24	75	72	4	147	1
	%	6.9	9.3	11.8	36.8	35.3		72.1	
I am satisfied with the mechanism for making suggestions to improve the system	N	94	44	33	16	18	2	34	2
	%	45.9	21.5	16.1	7.8	8.8		16.6	
The training I received was adequate	N	76	70	26	23	10	2	33	3
	%	37.1	34.1	12.7	11.2	4.9		16.1	
I am satisfied with the mechanism for identifying/reporting issues with the system	N	95	50	32	14	14	2	28	4
	%	46.3	24.4	15.6	6.8	6.8		13.6	
When the EHR system is down, the backup methods work adequately	N	90	58	30	17	9	2	26	5
	%	44.1	28.4	14.7	8.3	4.4		12.7	
Adequate resources were available when I was learning to use the EHR system	N	76	82	23	17	7	2	24.0	6
	%	37.1	40.0	11.2	8.3	3.4		11.7	
When I report problems with the system that need fixing, I receive adequate feedback	N	109	51	25	8	12	1	20.0	7
	%	53.2	24.9	12.2	3.9	5.9		9.8	
There was a campaign to introduce the EHR system prior to the implementation	N	89	75	23	11	7	2	18	8
	%	43.4	36.6	11.2	5.4	3.4		8.8	
Negative attitudes									
End-users should have been considered in the system design	N	2	6	21	56	120	5	176	1
	%	1.0	2.9	10.2	27.3	58.5		85.8	
It takes too much time to help others who do not know how to use the system	N	9	29	37	87	43	4	130	2
	%	4.4	14.1	18.0	42.4	21.0		63.4	
I am aware that problems with the EHR system have a direct impact on patient care	N	16	36	27	78	48	4	126	3
	%	7.8	17.6	13.2	38.0	23.4		61.4	
Using the EHR system takes longer than the paper-based system	N	75	81	13	18	18	2	36	4
	%	36.6	39.5	6.3	8.8	8.8		17.6	
Using EHRS raises stress levels among practitioners	N	94	65	18	19	7	2	26	5
	%	46.3	32.0	8.9	9.4	3.4		12.8	
The system makes me feel like I am no longer functioning as part of a team	N	73	85	21	15	10	2	25	6
	%	35.8	41.7	10.3	7.4	4.9		12.3	
The EHRS is considered to be an extra load at work	N	99	66	20	13	7	2	20	7
	%	48.3	32.2	9.8	6.3	3.4		9.7	

Media*n* = 1 strongly disagree; 2 disagree; 3 no opinion; 4 agree; 5 strongly agree.

Responses on the positive attitude scale indicated a strong consensus regarding essential factors such as EHRS usability, efficiency, and information quality. High endorsement was observed for usability items, with ease of use at 86.2% and physical comfort during system use at 87.2%. Efficiency was identified as a strength, with 95.1% of respondents agreeing that EHRS surpasses paper-based systems in this regard. Significant consensus was observed concerning information quality, with 93.6% agreement on the accuracy of information and 83.3% on the reliability of preventing information loss.

Responses to eight items in the training and support domain revealed significant dissatisfaction. The most significant levels of agreement regarding dissatisfaction were observed for: (1) inadequate support in addressing system issues (72.1%) and (2) low satisfaction with the process for submitting system improvement suggestions (16.6%). The lowest satisfaction ratings were linked to: (6) the availability of adequate resources during training (11.7%) and (8) the presence of an introductory campaign prior to system implementation (8.8%). These findings indicate substantial deficiencies in the training and support provided to EHRS users.

Negative attitudes, characterised by agreement indicating dissatisfaction, exhibited diverse levels of endorsement across seven items. The highest level of agreement, at 85.8%, was linked to the necessity for increased end-user involvement in system design. Subsequently, (2) concerns emerged regarding the time needed to support less experienced users (63.4%). Lower levels of agreement were noted for: (5) increased stress levels associated with EHRS use (12.8%), (6) feelings of disconnection from team dynamics (12.3%), and (7) perceptions of the system as an additional workload (9.7%).

### Inferential statistics

This section presents the results of inferential statistical analyses assessing the impact of demographic and experiential factors on EHRS end-user satisfaction. Given the ordinal nature of the data, non-parametric tests were conducted to determine significant differences across participant groups. A Mann–Whitney U test revealed that gender had no significant influence on any of the measured scales, including perceived usefulness (*p* = 0.559), positive attitude (*p* = 0.737), training and support (*p* = 0.113), and negative attitude (*p* = 0.338). Similarly, no significant differences were found based on EHRS use in the workplace, with all *p*-values exceeding 0.05 (see Table 8).

**Table 8:** Mann–Whitney U test results for gender and EHRS usage in the workplace.

Variable	Perceived usefulness	Positive attitude	Training and support	Negative attitude
Gender	0.559	0.737	0.113	0.338
Using EHRS at workplace	0.829	0.477	0.092	0.990

A Kruskal–Wallis test revealed that regional differences did not significantly influence EHRS satisfaction levels, with *p*-values exceeding 0.05 across all measured domains. In contrast, occupational role had a statistically significant impact on positive attitudes toward EHRS (*p* = 0.006), indicating that professional background influences user perceptions. Descriptive analysis, based on role-specific groupings in SPSS, showed that physicians and radiologists exhibited less favourable attitudes compared to laboratory technicians and chemists. This suggests that frontline clinical roles may face more complex interactions with EHRS, while technical staff benefit from more structured system use.

No significant occupational differences were found for perceived usefulness (*p* = 0.520), training and support (*p* = 0.166), or negative attitude (*p* = 0.964). Similarly, no significant effects were observed for years of job experience, EHRS experience, personal computer experience, or age, with *p*-values above 0.05 across all scales.

Overall, these findings indicate that occupation is the only demographic or experiential factor that significantly influences attitudes toward EHRS. This underscores the importance of tailoring implementation strategies and support mechanisms according to professional role to enhance system adoption and user engagement (see Table 9).

**Table 9:** Kruskal-Wallis test results for demographic and experiential factors.

Variable	Perceived usefulness	Positive attitude	Training and support	Negative attitude
Occupation	0.520	0.006	0.166	0.964
Region (Province)	0.726	0.197	0.610	0.221
Job experience	0.770	0.204	0.765	0.833
EHRS experience	0.309	0.373	0.303	0.570
Personal computer experience	0.598	0.590	0.804	0.218
Age	0.312	0.470	0.379	0.542

### Participant responses to open-ended questions

### Do you have any recommendations for the decision-makers to improve EHRS implementation?

As seen in Table 10 participants offered detailed recommendations for enhancing EHRS implementation in response to the open-ended question: “What recommendations do you have for decision-makers to improve EHRS implementation?” A total of 127 responses prior to implementation and 232 responses post-implementation were classified into ten primary themes through thematic analysis. Out of 232 post-implementation responses, only 188 pertained directly to the ten primary themes identified.

**Table 10:** Top ten recommendations for the decision-makers to improve the implementation of the EHRS?

Themes	Examples of responses	Frequency	Percentage
*Pre-implementation*			
Training	*“Increase training sessions”* *“Training should be during work hours.”* *“We need proper training.”* *“Provide training on site.”* *“Provide qualified trainers.”*	29	15%
Technical support	*“Technical support is very important.”* *“Provide ongoing technical support.”* *“Solve technical issues.”* *“Respond to issue reports.”*	25	13%
Involve the end-user	*“They should listen to the users’ requirements and suggestions.”* *“We want to participate in the system design.”* *“Involve those who will actually work with the system.”*	23	12%
Connectivity	*“Link all PHCs together.”* *“Connect the system to the Internet to exchange patient data with other organisations.”*	22	12%
Hardware	*“Update the old computers.”* *“Change our computers.”* *“Provide computers for everyone in the centre.”*	21	11%
Improvements	*“The current system needs some enhancements.”* *“The system needs ongoing improvement.”* *“Update the system on a regular basis.”*	18	10%
System security	*“I have concerns about the security of the system.”* *“System security is very important.”*	13	7%
Inclusiveness	*“The system should be comprehensive and serve all departments at the centre.”* *“Add all medical orders to help physicians.”* *“To benefit from the system, specific tools must be added.”*	13	7%
Technical requirements	*“Reduce the number of screens.”* *“Make it easier to switch from English to Arabic.”* *“Add an option to produce medical reports.”* *“Update the vaccinations form and all other forms in the system.”* *“Add tools to follow-up on patients with chronic diseases.”*	13	7%
CDSS	*“I hope they add CDSS to help us with medication.”* *“Better to include CDSS to reduce medication errors.”* *“Our problem is duplication in medication, so we need to have CDSS.”*	11	6%
Total		188	100%
*Post-implementation*			
Easy to use	*“Easy system”* *“Usability is important”*	26	20%
Infrastructure	*“Appropriate infrastructure”* *“Fast Internet”* *“Internet connection in the PHCs”* *“Infrastructure is very poor”*	23	18%
Hardware	*“We need new computers”* *“High performance computers and printers”* *“We ask them to change our old computers”*	15	12%
Staff involvement	*“Members of the centre should represent us during the implementation.”* *“They should hear our voice”*	14	11%
Training	*“Training, training, training”* *“Adequate training must be available for users”*	14	11%
Technical support	*“24-h support”* *“We need technical support all the time”* *“Technical support is important”*	11	9%
Consider PHC staff’ requirements	*“We have specific requirements that should be available in the EHRS”* *“Take our requirements into consideration”* *“We will use the system, and we have some requirements that must be available in the system”*	7	6%
Keenness for EHRS implementation	*“I asked the Ministry to implement the EHRS very soon”* *“We need it as soon as possible”*	6	5%
Secure system	*“Strong and secure system”* *“They should use anti-virus software to protect the system”* *“The IT department must test the system before implementing it to make sure it is protected against any breaches”*	6	5%
Link all PHCs together	*“It is better to link all PHCs with each other”* *“Information exchange between all PHCs”*	5	4%
Total		127	100%

### Pre-implementation recommendations

The predominant recommendation pertained to training, representing 20% (*n* = 26) of the responses. Participants highlighted the necessity of on-site training during work hours, facilitated by qualified trainers delivering appropriate instruction. Technical support emerged as the second most prevalent recommendation, accounting for 18% (*n* = 23), underscoring the significance of continuous assistance, prompt issue resolution, and readily available technical help. End-user involvement recommendations constituted 12% (*n* = 15), with participants advocating for increased inclusion in system design and implementation processes. Connectivity improvements and hardware upgrades each represented 11% (*n* = 14), highlighting the need to establish internet connectivity between PHCs and to provide updated computers. The least common recommendation, at 4% (*n* = 5), involved the incorporation of Clinical Decision Support Systems (CDSS) to mitigate medication errors.

### Post-implementation recommendations

Feedback following implementation identified new priorities, with usability recognized as the primary recommendation, accounting for 15% (*n* = 29) of responses. Participants highlighted the need for enhanced usability and user-friendliness of the system. Infrastructure development, encompassing high-speed internet and improved facilities, represented the second most prevalent theme (13%, *n* = 25). Hardware upgrades (12%, *n* = 23) and end-user involvement (12%, *n* = 21) remained significant considerations. Training was deemed essential (9%, *n* = 21), highlighting the need for sufficient and continuous programs. Additional significant themes encompassed technical support (9%, *n* = 18), consideration of staff requirements (6%, *n* = 11), and the assurance of a secure system (5%, *n* = 6). The recommendations with the lowest ranking pertained to linking PHCs to enhance information exchange (4%, *n* = 5).

## Discussion

This study evaluates EHRS implementation in PHCs by comparing pre- and post-implementation phases, providing insights for healthcare policymakers and project managers. Findings indicate significant perceived usefulness and readiness to adopt EHRS among end-users, highlighting its potential to enhance operational efficiency and patient care. Challenges including insufficient training, inadequate communication, and suboptimal infrastructure highlight essential areas that necessitate attention. The findings enhance the current literature by providing evidence-based insights from two separate phases of implementation within the same context, highlighting the necessity of customised strategies to overcome organisational and technological barriers. The study employs a unique methodology, utilising standardised data collection instruments, which facilitates replication in comparable contexts and enhances generalisability.

In this study, healthcare professionals demonstrated predominantly positive attitudes toward EHRS, with 96.6% expressing support for the system following its implementation. This is consistent with findings from King Khalid University Hospital in Saudi Arabia, where 89.5% of physicians and 87.9% of nurses reported satisfaction with EHRS, citing improvements in communication and workflow efficiency (6). Similarly, international studies have indicated that 81.1% of nurses hold favourable views toward EHR usage, recognising its role in enhancing clinical practice (4).

However, in contrast to a study conducted in Kuwait where 83% of medical receptionists and 89% of users overall expressed satisfaction due to system flexibility and usability (29) participants in the present study reported significant dissatisfaction related to training and technical support. This suggests that, despite general acceptance of EHRS, there remain critical gaps in infrastructure readiness and end-user support, particularly within the Saudi primary healthcare context.

Perceived usefulness and ease of use are identified as essential factors influencing user attitudes, as evidenced by this study and previous research. Clinicians worldwide have consistently regarded EHRS as instruments that improve communication, enable data retrieval, and enhance patient outcomes (6, 30). This study found significant agreement regarding the utility of EHRS, notably in cost reduction (93.7%), enhancement of document legibility (93.1%), and improvement of care coordination (92.2%). These findings are consistent with previous studies highlighting the significance of perceived usefulness (r = 0.52) and ease of use (r = 0.26) (30). However, the ongoing dissatisfaction with system usability in my study indicates a necessity for customised solutions that address specific organisational and cultural challenges.

The findings underscore the significance of training and support in the adoption of electronic health records (EHR). Studies in SA indicate generally positive attitudes toward training, with 74.2% of physicians and 71.7% of nurses expressing satisfaction (6). However, my findings reveal a significant contrast. PHC staff reported considerable dissatisfaction with the training mechanisms utilised in both pre- and post-implementation phases, with merely 16.6% indicating satisfaction with the adequacy of resources provided during training. The discrepancy may be due to the restricted availability of on-site training or the absence of customised training programs that meet the specific requirements of PHC staff. The findings align with global research demonstrating that insufficient training constitutes a significant obstacle to EHR adoption (5).

Leadership and organisational support are essential for cultivating favourable user attitudes during the implementation of electronic health records (EHR). Research indicates that robust management support improves readiness and satisfaction, with more than 92% of healthcare professionals in Saudi hospitals highlighting the significance of leadership (6). However, my findings identify deficiencies in this domain. Despite considerable enthusiasm for EHR implementation, 67.2% of PHC staff expressed disagreement with statements regarding their involvement in decision-making, and 67.2% reported feeling excluded from the implementation processes. The findings corroborate previous research highlighting the necessity of inclusive leadership strategies for effective EHR adoption (31).

Our findings contrast with studies that identify computer literacy as a key predictor of readiness and satisfaction with EHRS. Previous studies indicate a beneficial effect of computer experience on attitudes toward EHR adoption (12, 32); however, my research revealed no significant correlation between the duration of computer experience and individual readiness. This may be due to the elevated baseline computer literacy among PHC staff, with 86.9% possessing over five years of experience. The elevated computer proficiency noted in this study may have reduced its predictive impact, indicating that additional factors, such as the quality of training and the readiness of infrastructure, could be more significant.

The study identified technical infrastructure as a critical challenge, with participants frequently requesting improvements in connectivity and upgrades to hardware systems. Similar infrastructure-related barriers have been reported in both low-resource settings, such as Ethiopia, and high-income settings, such as the United Kingdom, where limitations in technical capacity have negatively affected EHR readiness (20, 33). These findings are also reflected in reports from other developed nations, including the United States, where gaps in infrastructure persist despite widespread EHR adoption (34). In contrast, primary healthcare centres (PHCs) in Lebanon reported greater readiness in terms of hardware availability, highlighting the contextual variability of infrastructural challenges across different healthcare systems (21).

The findings also emphasise the importance of perceived usefulness in driving EHR adoption. High satisfaction levels were observed among participants, particularly in relation to improved data accessibility, time efficiency, and workplace productivity. These outcomes are consistent with earlier research that positions perceived usefulness as a central determinant of positive attitudes toward EHR systems (35, 36). However, contrasting evidence exists in the literature, with some studies reporting decreased staff productivity following EHR implementation (37, 38). This discrepancy underscores the need for context-specific EHRS customisation, ensuring that the system meets the functional expectations of end-users and maximises its perceived benefits while minimising any negative impacts.

This study identified no significant correlation between participants’ computer experience and their satisfaction with EHRS implementation, consistent with previous research indicating diverse outcomes related to the influence of computer skills (39, 40). Some studies indicate a correlation between higher computer experience and increased satisfaction (41, 42), while others found no such association or noted a decrease in satisfaction over time (43, 44). This study found that physicians and radiologists exhibited less positive attitudes than laboratory technicians and chemists. This aligns with the findings of Khalifa and Alswailem (40) and Bossen, Jensen (45), but contrasts with research indicating higher satisfaction levels among nurses and administrative staff (46). In contrast, other research conducted by Secginli and Erdogan (47) found no significant differences in attitudes among various occupations. The findings underscore the complexity of factors affecting satisfaction, indicating that user attitudes are more significantly influenced by systemic and organisational elements than by individual traits such as experience or profession. This emphasises the necessity for further research across various user groups.

### Study limitations and implications

This study presents several limitations that warrant acknowledgement. One limitation of the study is the relatively high proportion of administrative staff among the respondents, which may influence the generalisability of findings regarding system usability and readiness, particularly from a clinical standpoint. In addition, while key demographic variables such as age, gender, and job role were collected, the questionnaire did not include items on participants’ educational level or nationality factors which could provide further insight given the diversity of the Saudi healthcare workforce. These will be considered in future research to enhance the contextual understanding of EHRS adoption. Furthermore, the dependence on self-reported data may lead to bias, as participants’ perceptions and responses might be affected by social desirability or inaccuracies in recall. The study was conducted exclusively within PHCs in SA, which restricts the generalisability of the findings to other healthcare environments or countries characterised by differing organisational, cultural, and technological frameworks. Third, the study lacked the inclusion of objective performance measures, such as system usage logs or patient outcomes, which could offer a more thorough assessment of EHRS implementation. The cross-sectional design of the pre- and post-implementation phases provides a snapshot of user attitudes and readiness; however, it fails to account for longitudinal changes in perceptions and experiences over time. Future research may overcome these limitations by incorporating objective data, broadening the scope to encompass various healthcare settings, and employing longitudinal methods to evaluate the changing effects of EHRS implementation.

The findings hold substantial implications for practice, policy, and research. The findings highlight the necessity for improved training programs, strong technical support, and increased staff involvement during the implementation of EHRS to enhance system acceptance and usability. Policymakers can utilise these insights to create comprehensive frameworks for the effective deployment of EHRS, especially in resource-limited settings. This study provides a basis for longitudinal evaluations of health IT initiatives, promoting additional investigation into the factors that affect the success of EHRS over time.

The study’s strengths lie in its comparative design across two implementation phases and the utilisation of validated instruments, which bolster the reliability of the findings. Nevertheless, constraints such as dependence on self-reported data and concentration on a singular context may restrict the generalisability of the findings. Future research should address these limitations by integrating objective measures and broadening the scope to encompass various healthcare settings. This study enhances the current literature on EHRS implementation through a comprehensive analysis of user attitudes, readiness, and system effectiveness in Saudi PHCs. The text emphasises the importance of addressing contextual challenges, including training adequacy, leadership involvement, and infrastructure readiness, to facilitate successful adoption. The study identifies universal and context-specific factors influencing EHRS implementation by comparing its findings with previous research, providing insights for policymakers and healthcare administrators seeking to enhance EHRS adoption across different settings.

### What this study adds

- This study provides a comparative analysis of the pre- and post-implementation phases of EHRS in PHCs, offering practical insights to inform the planning and execution of similar large-scale initiatives by project managers and policymakers.
- The findings underscore critical lessons for IT project managers and healthcare decision-makers, particularly the pivotal role of end-user engagement, structured training programmes, and infrastructure preparedness in facilitating successful EHRS adoption.
- A key contribution of this study lies in its examination of two distinct phases within the same organisational setting, thereby enabling a longitudinal perspective on healthcare staff attitudes and organisational readiness over time.
- The use of validated and replicable data collection instruments enhances the study’s methodological rigour and provides a reliable framework for future research on EHRS adoption and its influencing factors in comparable healthcare environments.

## Conclusion

This study emphasises the dual aspects of opportunities and challenges associated with the implementation of Electronic Health Record Systems in Primary Health Centres. A substantial majority of participants (97.7%) indicated a readiness to support the system, highlighting advantages such as increased productivity, enhanced data accuracy, improved accessibility, and decreased medication errors and administrative burdens. However, notable obstacles persist. Significant challenges encompass limited staff engagement in decision-making, poor communication among project teams and PHCs, and organisational unpreparedness, especially regarding training and support, with 63.7 and 69.2% of participants expressing dissatisfaction, respectively. Moreover, technological readiness was impeded by inadequate infrastructure and obsolete hardware. Participants’ generally favourable attitudes toward EHRS highlight the system’s perceived potential to enhance patient care and operational efficiency, despite existing barriers. Dissatisfaction with training and technical support underscores the necessity for targeted interventions in these domains, alongside the incorporation of advanced tools to address varied professional requirements. The findings provide valuable insights for policymakers and healthcare administrators to improve the adoption of EHRS and enhance outcomes in comparable healthcare environments.

## Data Availability

The raw data supporting the conclusions of this article will be made available by the authors, without undue reservation.
